# Caveolin-1 Knockdown Decreases SMMC7721 Human Hepatocellular Carcinoma Cell Invasiveness by Inhibiting Vascular Endothelial Growth Factor-Induced Angiogenesis

**DOI:** 10.1155/2020/8880888

**Published:** 2020-06-27

**Authors:** Zhi-Bo Zhang, Zheng Shi, Lan-Fang Yang, Hong-Bin Gao

**Affiliations:** Department of Hepatopancreatobiliary Surgery, The First Affiliated Hospital, Fujian Medical University, Fuzhou 350004, China

## Abstract

**Background:**

Recently, several studies have demonstrated that caveolin-1 overexpression is involved in apoptosis resistance, angiogenesis, and invasiveness in hepatocellular carcinoma (HCC). However, the mechanisms underlying caveolin-1-mediated tumor progression remain unclear. *Methodogy*. Lentiviral vectors were used to construct caveolin-1 small interfering RNA- (siRNA-) expressing cells. Secreted VEGF levels in SMMC7721 cells were evaluated by enzyme-linked immunosorbent assay (ELISA). SMMC7721 cell proliferation, cycle, apoptosis, and invasiveness were detected by MTT, flow cytometry, Annexin V-FITC/PI, and invasion assay, respectively. Phospho-eNOS levels in human umbilical vein endothelial cells (HUVECs) cocultured with SMMC7721 cell supernatants were analyzed by Western blot. Capillary-like tubule formation assay was performed to analyze endothelial tubular structure formation in HUVECs treated with supernatants from caveolin-1 siRNA-expressing SMMC7721 cells. SMMC7721 implantation and growth in nude mice were observed. Angiogenesis *in vivo* was analyzed by immunohistochemical angiogenesis assay.

**Results:**

Caveolin-1 siRNA-expressing SMMC7721 cells secreted reduced levels of VEGF. Caveolin-1 RNAi also caused an inhibition of SMMC7721 cell proliferation and cell cycle progression that was accompanied by increased apoptosis. Supernatants from caveolin-1 siRNA-expressing SMMC7721 cells inhibited cell cycle progression and decreased phospho-eNOS levels in HUVECs. Endothelial tubular structure formation in HUVECs treated with supernatants from caveolin-1 siRNA-expressing SMMC7721 cells was considerably reduced. Caveolin-1 siRNA-expressing SMMC7721 cells also showed reduced tumorigenicity and angiogenesis induction *in vivo*.

**Conclusion:**

Our results reveal a novel mechanism, whereby caveolin-1 positively regulates human HCC cell invasiveness by coordinating VEGF-induced angiogenesis.

## 1. Introduction

Caveolin-1, an essential constituent of plasma membrane invaginations called caveolae, is known to be involved in cancer progression and angiogenesis. Numerous studies have indicated that the role of caveolin-1 expression depends on the cell type and the stage of the cancer in question. Although caveolin-1 is known to act as a tumor suppressor in cases of pulmonary adenocarcinomas and sarcomas [1, 2], several recent studies found that caveolin-1 overexpression was associated with invasion and angiogenesis. In esophageal squamous cell carcinomas and pancreatic ductal adenocarcinomas, caveolin-1 overexpression correlates with a high histological grade, an advanced tumor stage and metastasis [3, 4]. Bailey and Liu [5] demonstrated that caveolin-1 expression was upregulated during the epithelial-to-mesenchymal transition; furthermore, once expressed, caveolin-1 greatly increased cancer cell adhesion. Additionally, Joo et al. [6] reported that increased caveolin-1 expression and microvessel density (MVD) correlated with metastasis and poor prognosis in renal cell carcinoma patients. These data imply that caveolin-1 might mediate tumor invasiveness by stimulating angiogenesis.

When involved in tumor growth and metastasis, angiogenesis is considered to be a pathological event [7]. Multiple studies have demonstrated that vascular endothelial growth factor (VEGF) is a key positive regulator of angiogenesis [8]. Previous studies reported that VEGF [9] was associated with hepatocellular carcinoma (HCC) progression and angiogenesis. However, studies concerning the relevance of caveolin-1 in VEGF-induced angiogenesis have been predominately focused on endothelial cells [10–13]. Therefore, the connections and interactions between caveolin-1 expression, cancer progression, and angiogenesis in HCC remain unknown.

These previous findings regarding the function of caveolin-1 in cancer led us to hypothesize that caveolin-1 could induce VEGF expression in HCC cells, subsequently enhancing tumor angiogenesis and promoting HCC invasiveness. Our previous study found that the SMMC7721 human HCC cell line had higher caveolin-1 expression accompanied by higher invasive ability than other human HCC cell lines [14]. Therefore, using RNA interference (RNAi), we investigated the effect of caveolin-1 on the SMMC7721 human HCC cell line and evaluated its role in invasiveness and angiogenesis.

## 2. Materials and Methods

### 2.1. Materials and Reagents

Dulbecco's modified Eagle's medium (DMEM) and fetal bovine serum (FBS) were purchased from Gibco. Lipofectamine 2000 and Trizol were purchased from Invitrogen. The restriction enzymes Age I and EcoR I were purchased from New England BioLabs. SYBR®PrimeScript™ RT-PCR Kits were purchased from TaKaRa. Human VEGF Quantikine enzyme-linked immunosorbent assay (ELISA) kits were purchased from R & D Systems. ECL Western blotting detection reagent was purchased from Amersham Biosciences. BD Matrigel Matrix and Growth Factor-Reduced (GFR) BD Matrigel Matrix were purchased from BD Biosciences. Twenty-four-well Millicells with 8 *μ*m Transwell chambers were purchased from Millipore. Annexin V-FITC/PI kits were purchased from Sigma. Nude mice were purchased from Shanghai Slac Laboratory Animal Co. Ltd.

The lentivirus packaging mix (GenScript siRNA expression vector pGCSIL-Green fluorescent protein (GFP), pHelper 1.0 and pHelper 2.0), enhanced infection solution (ENi.S), and polybrene were purchased from GeneChem; GeneChem also synthesized the oligonucleotide primers used in this study. The polyclonal rabbit anti-caveolin-1 antibody and mouse anti-GAPDH and anti-VEGF monoclonal antibodies were purchased from Santa Cruz Biotechnology. The monoclonal mouse anti-CD34 antibody was purchased from NeoMarkers. The monoclonal rabbit anti-eNOS and anti-phospho-eNOS antibodies were purchased from Cell Signaling. Recombinant Human VEGF (treated with 10 ng/ml) [13] was purchased from R & D Systems.

### 2.2. Cell Culture

The SMMC7721 human HCC cell line and 293T cell line (Shanghai Institute of Cell Biology, Chinese Academy of Sciences, Shanghai, China) were cultured in DMEM supplemented with 10% FBS; cells used in the study were passaged fewer than 20 times. Control SMMC7721 (CON, no infection), negative control SMMC7721 (NC, infected with empty pGCSIL-GFP vector), and knockdown SMMC7721 (KD, infected with plasmid encoding caveolin-1 siRNA-pGCSIL-GFP) cells were incubated in a 37°C incubator with 5% CO_2_.

Primary cultured human umbilical vein endothelial cells (HUVECs) were obtained from consenting healthy patients as described previously [15], with minor modifications; all institutional guidelines were followed. HUVECs were grown in DMEM supplemented with 20% FBS and endothelial cell growth supplement.

For construction of lentiviral vector (LV) for caveolin-1 small interfering RNA and DNA plasmid, we have the following.

Small interfering RNAs (siRNAs) targeting the human caveolin-1 gene (Genebank accession number: NM_001753) were designed by Shanghai GeneChem, Co. Ltd, China. Different siRNAs were screened by cotransfection with a human caveolin-1 cDNA plasmid into 293T cells with Lipofectamine 2000. The optimal siRNA sequence against human caveolin-1 (5′-CTTCAGACCCGCCAACAAA-3′) was then cloned into the pGCSIL-GFP plasmid that encodes an HIV-derived LV. Lentivirus preparations were produced by the Shanghai GeneChem Co. Ltd, China. The resulting LV that contained human caveolin-1 siRNA was named caveolin-1-siRNA-LV, and its sequence was confirmed by quantitative real-time PCR (qRT-PCR) and sequence analysis. SMMC7721 cells were infected with the caveolin-1-siRNA-LV lentivirus by adding lentivirus to the cell culture at a multiplicity of infection (MOI) of approximately 20; ENi.S and polybrene were also added to increase virus infectivity. Control cells were infected with the negative control (NC) lentivirus or not infected (CON). After 5 days of infection, the knockdown efficiency was assessed using qRT-PCR and Western blot. The lentiviral DNA plasmid for human caveolin-1 (pGC-FU-caveolin-1) was constructed in our previous study [16].

### 2.3. qRT-PCR

Total RNA was collected using Trizol; cDNA was reverse-transcribed using a SYBR®PrimeScript™ RT-PCR Kit. Caveolin-1 mRNA expression levels were analyzed using qRT-PCR performed on a LightCycler instrument. The PCR conditions consisted of 45 cycles of denaturation for 5 s at 95°C, annealing for 20 s at 60°C, and primer extension for 15 s at 72°C. The primers were as follows for human caveolin-1: forward, 5′ CTGAGCGAGAAGCAAGTG 3′ and reverse, 5′ AGAGAGAATGGCGAAGTAAATG 3′). For human *β*-actin, the primers were as follows: forward, 5′ GTGGACATCCGCAAAGAC 3′ and reverse, 5′ AAAGGGTGTAACGCAACTA 3'. All qRT-PCR results are expressed as fold changes in mRNA expression with respect to the control cells. Target gene expression was normalized to the expression of the housekeeping gene for each sample. Data were analyzed using the 2^−ΔΔCt^ method. All reactions were performed in triplicate. The efficacy of 3 target sequences was evaluated, and the sequence with the best efficacy was selected and utilized in subsequent experiments.

### 2.4. 3-(4,5-Dimethylthiazol-2-yl)-2,5-diphenyltetrazolium Bromide (MTT) Cell Proliferation Assay

Following infection with the KD or NC lentivirus for 120 h, cells were incubated with 3 mg/ml MTT for 4 h at 37°C. MTT/formazan was extracted through an overnight incubation at 37°C with 100 *μ*l extraction buffer [20% SDS, 50% formamide (pH 4.7), 0.02% acetic acid and 0.025 mol/L HCl]. Optical densities at 570 nm were measured using extraction buffer as a blank.

### 2.5. Annexin V-Fluorescein Isothiocyanate (FITC)/Propidium Iodide (PI) Cell Apoptosis Assay

The Annexin V-FITC/PI assay for caveolin-1 siRNA-expressing SMMC7721 cells was performed according to the manufacturer's instructions. A total of 1 × 10^6^ cells were washed with binding buffer and resuspended in 100 *μ*l binding buffer and Annexin V-FITC (titer from 0.1–1.0 *μ*g). After incubating the cells at room temperature for 10 minutes, 400 *μ*l of binding buffer containing 1 *μ*l PI was added; cells were analyzed by flow cytometry within one hour.

### 2.6. Flow Cytometric Cell Cycle Analysis

Flow cytometric analysis of SMMC7721 cells that expressed caveolin-1 siRNA and HUVECs cocultured with SMMC7721 cell supernatants was performed using a Becton Dickinson FACStarPLUS instrument. GFP was detected in cells suspended in fluorescence-activated cell sorting buffer composed of 0.5% bovine serum albumin in phosphate-buffered saline (PBS) supplemented with 0.05% sodium azide. The percentage of live, GFP-positive cells was calculated.

### 2.7. Invasion Assay

Invasion assays were performed using a Millicell chamber system with 0.25 mg/ml BD Matrigel-precoated polycarbonate membranes. While the bottom chambers were filled with DMEM supplemented with 10% FBS as a chemoattractant, the top chambers contained serum-free DMEM. Cells (5 × 10^4^ per well) were added to the top chamber and incubated for 24 h and 48 h at 37°C in a 5% CO_2_ incubator. Three independent experiments were performed for each set. The cells that migrated through the Matrigel and adhered to the bottom of the membrane were fixed and stained with crystal violet. Cells in ten microscopic fields at a 100x magnification were counted and averaged.

### 2.8. Secreted VEGF Quantitation by ELISA

Supernatants from caveolin-1 siRNA-expressing SMMC7721 cells were prepared as described previously [17]. To quantitate secreted human VEGF levels, ELISAs were performed according to the manufacturer's protocol. VEGF levels were evaluated by final unit cell numbers (pg VEGF/10^5^ cells/24 h) because of differences in the seeding efficiency between experiments.

### 2.9. Western Blot

After being cocultured with SMMC7721 cell supernatants for 24 h or 48 h, HUVECs were rinsed with PBS and lysed with sodium dodecyl sulfate-polyacrylamide gel electrophoresis (SDS-PAGE) protein loading buffer containing 5% 2-mercaptoethanol. Cell lysates with equal amounts of total protein were then separated on 10% SDS-polyacrylamide gels with a 12% polyacrylamide gel and transferred onto nitrocellulose membranes. The resulting Western blots were incubated with 5% bovine serum albumin (BSA) for 1.5 h and then incubated overnight at 4°C with monoclonal rabbit antibodies directed against caveolin-1, eNOS, or phospho-eNOS (ser-1177) (1 : 1000). Detection of glyceraldehyde-3-phosphate dehydrogenase (GAPDH) using an anti-GAPDH antibody served as an internal control. The blots were then washed 3 times with 0.05% Tween-20 in TBS and incubated with peroxidase-conjugated anti-goat secondary antibody (1 : 5000) for 2 h at room temperature. After further washing, the blots were developed using ECL Western blotting detection reagent, and immunoreactive proteins were visualized on Kodak X-Omat film. The siRNA knockdown conditioned medium efficiency was also analyzed using this approach.

### 2.10. Capillary-like Tubule Formation Assay and Immunocytochemistry

The formation of capillary-like tubular structures was assessed in Matrigel-coated multiwell plates as described previously [18]. HUVECs were seeded at a density of 1 × 10^5^ cells/ml with SMMC7721 cell supernatants in GFR BD Matrigel matrix-coated 24-well plates and incubated at 37°C for 0–24 h. The formation of tubular structures was examined using an Olympus BX41 microscope. After 24 h of culture, the cells were fixed with 4% paraformaldehyde for 20 min, blocked with 5% goat serum for 20 min, and then stained with anti-CD34 antibodies for 2 h at 37°C. After a second incubation with a two-step IHC detection reagent (PV-6002) (ZSGB-BIO, Beijing, China), reaction products were visualized by immersing the slides in a solution 3,3′diaminobenzidine tetrachloride containing substrate (ZLI-9032) (ZSGB-BIO, Beijing, China). Finally, the slides were counterstained with Mayer's hematoxylin.

### 2.11. SMMC7721 Implantation and Growth in Nude Mice

To investigate the tumorigenicity of SMMC7721 cells infected with caveolin-1-siRNA-LV lentivirus, exponentially growing cells were harvested. Briefly, cells were washed with PBS and then exposed to 0.25% trypsin/0.02% EDTA for a short period of time. The trypsin was neutralized by culture medium, and the cells were washed once with serum-free medium (SFM). A total of 5 × 10^6^ cells from each group were suspended in 100 *µ*l SFM; cell preparations were then inoculated subcutaneously into the spleens of 6-week-old athymic male nude mice. Six weeks after inoculation, all mice were sacrificed. The size of tumors in the spleen and liver was measured with calipers. The tumor volume (mm^3^) was estimated using the formula *L* × *W*^2^ × 0.52, where *L* is the length and *W* is the width. The number of tumors in the liver was also counted. Tumors were dissected and weighed individually prior to being fixed in 10% formalin.

### 2.12. Immunohistochemical Angiogenesis Assay

To determine whether differences in angiogenesis *in vivo* corresponded to altered production of the growth factors expressed by these cells *in vitro*, tumor sections were analyzed by immunohistochemical staining. Tumor cells were stained with anti-VEGF and anti-CD34 antibodies. MVD values were determined by examining CD34-stained slides. Individual microvessels were counted in the areas of highest vascularity in five selected microscopic fields examined at a magnification of 200x. Any brown-stained endothelial cells or clusters that were separated from nearby microvessels were counted. The presence of a vascular lumen was not necessary to identify a microvessel. Large anastomosing sinusoidal vessels were counted as a single vessel. Large vessels with thick muscular walls were excluded. The microvessel count was expressed as the mean number of vessels in the selected area.

### 2.13. Statistical Analysis

Each experiment was performed at least 3 times; data shown represent the mean ± SD where applicable. Statistically significant differences between groups in each assay were determined using a paired *T* test or an ANOVA followed by a post hoc Dunnett's *t*-test. A probability value of <0.05 was considered statistically significant.

## 3. Results

### 3.1. Human Caveolin-1 mRNA and Protein Levels Were Significantly Reduced following Caveolin-1 siRNA-LV Infection

**Figure 1:**
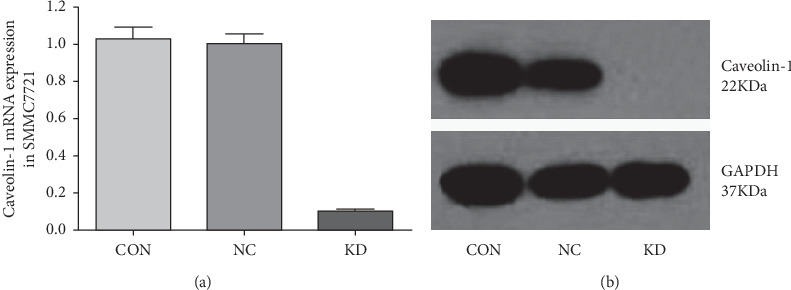
The mRNA and protein levels indicate the knockdown efficiency of caveolin-1 siRNA. (a) Caveolin-1 mRNA expression as detected by qRT-PCR. Its levels were knocked down by approximately 90%. (b) Caveolin-1 protein expression as detected by Western blot. Its levels were reduced by approximately 100%.

In infected SMMC7721 cells, the LV expression cassette enabled the permanent expression of GFP and caveolin-1 siRNA. GFP expression and the percentage of GFP-expressing cells were determined by flow cytometric analysis. The transduction efficiencies of the caveolin-1 siRNA-LV and the NC lentivirus were approximately 96%. After 4 d of infection with the caveolin-1 siRNA-LV, caveolin-1 mRNA and protein levels were knocked down by 90% (Figure 1(a)) and 100% (Figure 1(b)), respectively.

### 3.2. Caveolin-1 siRNA-Expressing SMMC7721 Cells Secreted Reduced Levels of VEGF

**Figure 2:**
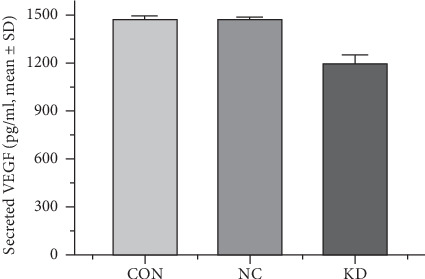
Quantitation of secreted VEGF by ELISA. VEGF secretion from control (CON), negative control (NC), and caveolin-1 siRNA-LV (KD) groups was (1465 ± 26.21), (1466 ± 17.62), and (1193 ± 50.24) pg/ml, respectively (KD versus CON, *P*=0.011; KD versus NC, *P*=0.010).

We evaluated VEGF secretion by analyzing the supernatants of SMMC7721 cells in the CON, NC, or KD groups. Among the three groups, VEGF levels in the KD group (1193 ± 50.24 pg/ml) were significantly reduced compared to the CON (1465 ± 26.21 pg/ml) and NC (1466 ± 17.62 pg/ml) groups (Figure 2).

Caveolin-1 siRNA-expressing SMMC7721 cells exhibited decreased cell proliferation and cell cycle progression that was associated with increased apoptosis and reduced cell invasiveness.

**Figure 3:**
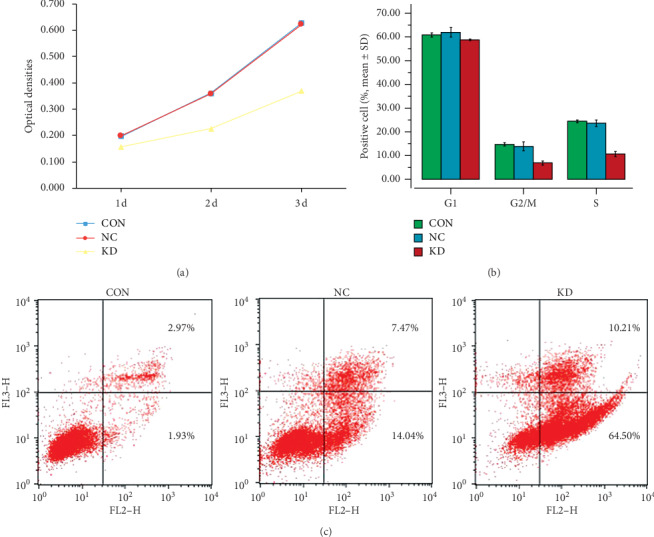
Cell proliferation (a), cell cycle progression (b), and apoptosis (c) of SMMC7721 cells in the control (CON), negative control (NC), and caveolin-1 siRNA-LV (KD) groups were detected by the MTT assay, flow cytometric analysis, and the Annexin V-FITC/PI assay, respectively. (a) Cell proliferation in the KD group was significantly decreased at days 3 and 5 after infection compared to the CON or the NC group (*P* < 0.01). (b) At day 3, the proportions of KD cells in the G2/M and S stages were significantly reduced compared to the CON or the NC group (*P* < 0.01). (c) At 24 h, apoptosis in the KD group was significantly increased compared to the CON or the NC group (*P* < 0.01).

SMMC7721 cells in the CON, NC or KD groups were analyzed for cell proliferation using the MTT assay (Figure 3(a)) and cell cycle status by flow cytometric analysis (Figure 3(b)). The data indicated that caveolin-1 knockdown significantly decreased cell proliferation and cell cycle progression.

The Annexin V-FITC/PI assay revealed that caveolin-1 knockdown promoted SMMC7721 cell apoptosis (Figure 3(c)).

**Figure 4:**
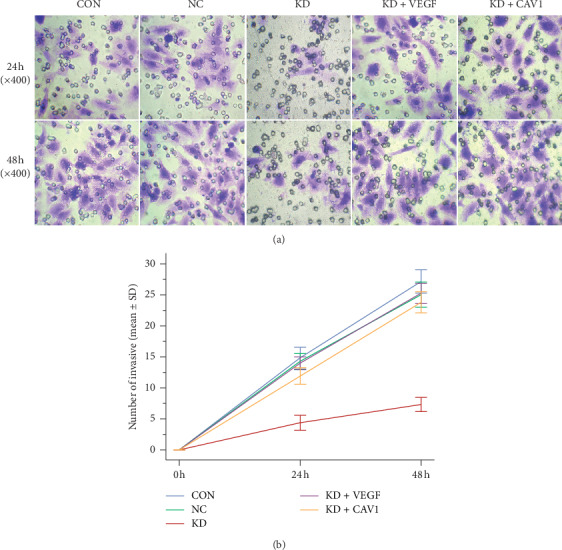
Invasion assays were performed for SMMC7721 cells in the control (CON), negative control (NC), and caveolin-1 siRNA-LV (KD) groups using a Millicell chamber system with BD Matrigel. (a) The number of invasive cells in the KD group was significantly reduced at 24 h and 48 h; invasiveness was significantly restored following supplementation with recombinant human VEGF (KD + VEGF) or infection with the pGC-FU-caveolin-1 DNA plasmid LV (KD + CAV1). (b) The invasiveness difference between the group KD and the CON, NC, KD + VEGF, and KD + CAV1 groups was quantitated by counting the number of cells that migrated to the lower level of the membrane in ten microscopic fields at 100x magnification and averaging the values. The data are presented as the mean ± SD from three independent experiments; in each experiment, conditions were performed in duplicate. At 24 h and 48 h, KD versus CON, *P* ≤ 0.001; KD versus NC, *P* ≤ 0.001; KD + VEGF versus KD, *P* ≤ 0.001; KD + CAV1 versus KD, *P* ≤ 0.001; KD + VEGF versus KD + CAV1, *P* > 0.05).

Invasion assays were performed to elucidate the effect of caveolin-1 downregulation on SMMC7721 cell invasiveness. The number of invasive cells in the KD group was significantly lower compared to the CON or KD groups at both 24 h and 48 h. However, invasiveness was restored when KD cells were supplemented with recombinant human VEGF (KD + VEGF) or infected with the pGC-FU-caveolin-1 DNA plasmid LV (KD + CAV1) (Figure 4). The results imply that SMMC7721 cell invasiveness is positively regulated by caveolin-1 expression by inducing VEGF expression.

### 3.3. Supernatants from Caveolin-1 siRNA-Expressing SMMC7721 Cells Inhibited Cell Cycle Progression and Decreased Phospho-eNOS Levels in HUVECs

**Figure 5:**
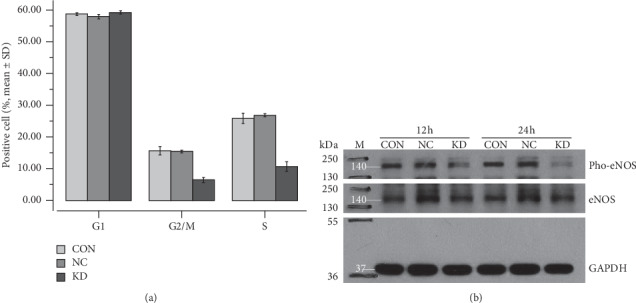
Cell cycle progression and phospho-eNOS levels in HUVECs incubated with supernatants from control (CON), negative control (NC), or caveolin-1 siRNA-LV (KD) SMMC7721 cells were detected by flow cytometric analysis and Western blot assay, respectively. (a) At day 3, the proportions of KD cells in the G2/M and S stages were considerably reduced compared to the CON or the NC group (*P* < 0.01). (b) At 12 h and 24 h phospho-eNOS (ser1177) levels in HUVECs treated with KD group supernatants were considerably decreased compared to the CON or the NC group (*P* < 0.01). However, the total eNOS expression in HUVECs in each group was not significantly different (*P* > 0.05).

Flow cytometric analyses and Western blot assays were performed to confirm the effect of supernatants from caveolin-1 siRNA-expressing SMMC7721 cells on cell cycle progression and phospho-eNOS levels in HUVECs. Treatment with supernatants from caveolin-1 siRNA-expressing SMMC7721 cells significantly decreased HUVEC cell cycle progression compared to the CON or KD groups (Figure 5(a)). Additionally, phosphorylation of eNOS at ser1177 in HUVECs was significantly decreased following treatment with supernatants from caveolin-1 siRNA-expressing SMMC7721 cells compared to the CON or KD groups (Figure 5(b)).

### 3.4. Considerably Reduced Endothelial Tubular Structure Formation in HUVECs Treated with Supernatants from Caveolin-1 siRNA-Expressing SMMC7721 Cells

**Figure 6:**
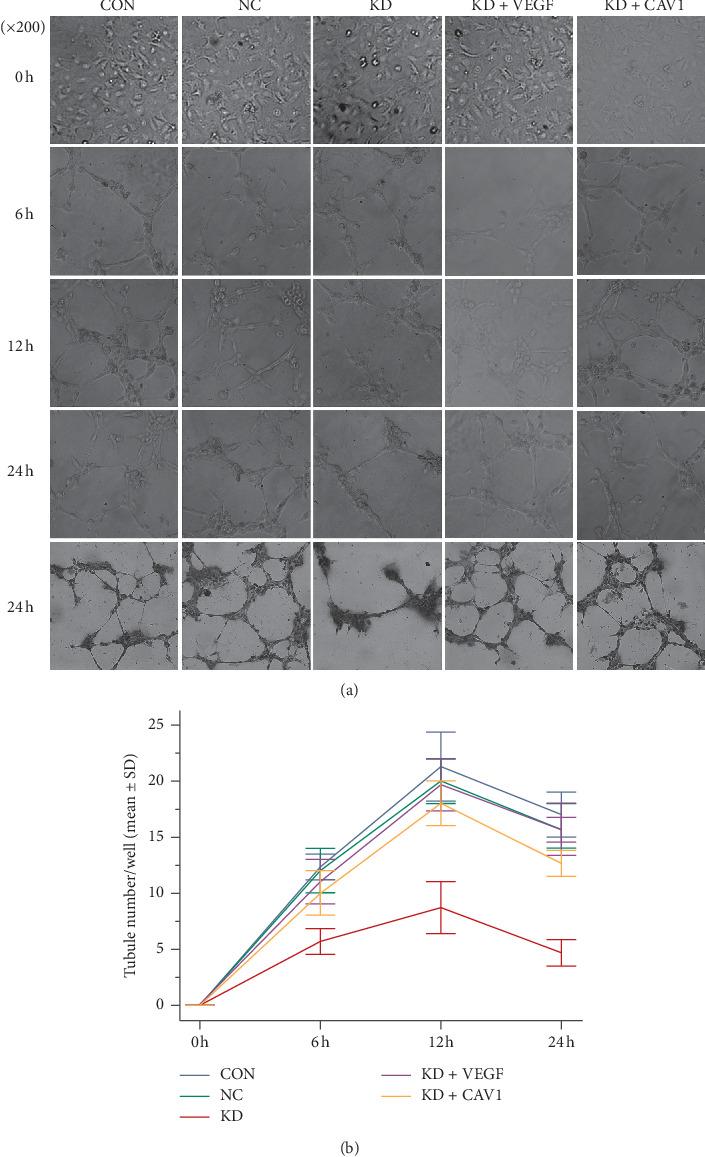
Supernatants from caveolin-1 siRNA-expressing SMMC7721 cells significantly inhibited the formation of endothelial tubular structures. (a) Bright-field views were taken using an Olympus BX40 fluorescent microscope equipped with a photometric CCD camera. Note that compared to cells treated with supernatants from control (CON) and negative control (NC) SMMC7721 cells at 6 h, 12 h, and 24 h fewer, capillary-like tubules developed in cells treated with supernatants from cells with knocked down caveolin-1 (KD). Capillary-like tubule formation was recovered following the addition of recombinant human VEGF (KD + VEGF); capillary-like tubule formation was also rescued if cells were treated with supernatants from KD cells subsequently infected with pGC-FU-caveolin-1 DNA plasmid LV (KD + CAV1). Immunocytochemical labeling of CD34 was performed at 24 h to further examine capillary-like tubule formation. (b) The difference in capillary-like tubule formation between cells treated with KD-derived supernatants and cells treated with supernatants from CON, NC, KD + VEGF, or KD + CAV1 SMMC7721 cells was quantitated. Capillary-like tubular structures were scored by counting the number of tubules in five microscopic fields at a magnification of 200x; values from each well were then averaged. Data represent the mean ± SD from three independent experiments; all samples were run in duplicate. At 6 h, 12 h, and 24 h, the results were as follows: KD versus CON, *P* ≤ 0.001; KD versus NC, *P* ≤ 0.001; KD + VEGF versus KD, *P* ≤ 0.001; KD + CAV1 versus KD, *P* ≤ 0.001; KD + VEGF versus KD + CAV1, *P* > 0.05).

HUVECs were cultured with supernatants from CON, NC, or KD SMMC7721 cells to evaluate the formation of endothelial tubular structures. Fewer capillary-like tubules developed the GFR BD Matrigel Matrix in HUVECs treated with KD group supernatants compared to HUVECs treated with CON- or NC-derived supernatants; these results were observed at 6, 12, and 24 h of incubation. The number of tubules significantly increased in HUVECs treated with supernatants from KD cells following addition of recombinant human VEGF (KD + VEGF); similar increases in HUVEC tubule formation were also observed when HUVECs were treated with KD cells that were subsequently infected with the pGC-FU-caveolin-1 DNA plasmid LV (KD + CAV1). MVD analysis through immunocytochemical detection of CD34, which was performed at 24 h, further confirmed these results (Figure 6).

### 3.5. Caveolin-1 siRNA-Expressing SMMC7721 Cells Showed Reduced Tumorigenicity and Angiogenesis Induction *In Vivo*

**Figure 7:**
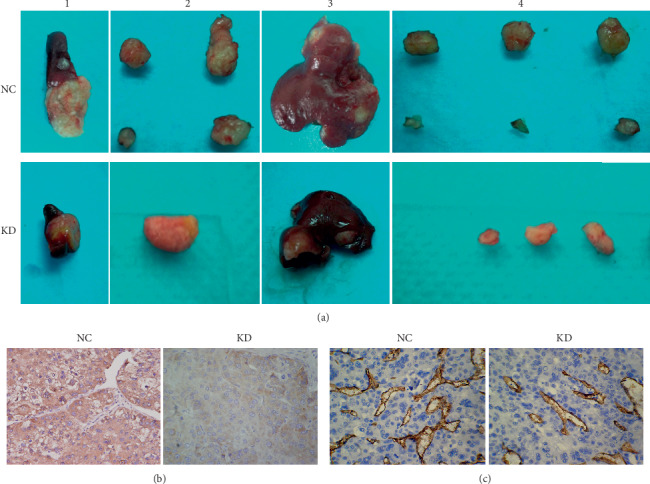
Comparison of SMMC7721 cell line tumorigenicity and induction of angiogenesis with caveolin-1 siRNA or negative control *in vivo*. (a) The final tumors were photographed immediately after dissection. Their relative sizes in the spleen (1, 2) are shown against a ruler. The maximal tumor volume in the caveolin-1 siRNA-LV (KD) and negative control (NC) groups was (347.2 ± 101.7) and (735.6 ± 159.1) mm^3^, respectively (*P* < 0.01). The numbers of metastatic tumors in the liver (3, 4) for the caveolin-1 siRNA-LV (KD) and negative control (NC) groups were (0.9 ± 0.3) and (3.5 ± 1.9), respectively (*P* < 0.01). (b) Immunohistochemical staining of VEGF in tumor sections viewed at 400x magnification; VEGF is stained dark brown. Compared to the NC group, the VEGF levels in the KD group were significantly lower (*P* < 0.01). (c) Immunohistochemical staining of CD34 in tumor sections viewed at 400x magnification; CD34 is stained dark brown. Compared to the NC group, the MVD of the KD group was significantly lower (*P* < 0.01).

The tumors dissected from the spleen and liver six weeks after injection are presented in Figure 7(a). After 6 weeks, caveolin-1 siRNA-expressing SMMC7721 cells generated an average splenic tumor mass that was approximately 2 times less compared to negative control SMMC7721 cells. Additionally, the average metastatic tumor quantity in the liver was approximately 5 times less in mice treated with caveolin-1 siRNA-expressing SMMC7721 cells compared to mice treated with negative control SMMC7721 cells.

Tumor sections were analyzed by immunohistochemistry and stained for VEGF to identify *in vivo* differences in angiogenesis caused by decreased caveolin-1 expression. VEGF levels were lower in caveolin-1 siRNA-expressing SMMC7721 cells compared to negative control SMMC7721 cells in the spleen (Figure 7(b)). Although CD34 staining indicated that angiogenesis occurring in all tumors, smaller and thinner blood vessels were found in caveolin-1 siRNA-expressing SMMC7721 tumor sections compared to negative control tumors in the spleen (Figure 7(c)). These data suggest a positive correlation between caveolin-1 expression and angiogenesis *in vivo.*

## 4. Discussion

Most studies indicate that caveolin-1 promotes cell polarization and directional migration through the coordination of Src kinase and Rho GTPase signaling; in turn, these cellular processes play key roles in many pathological processes, including angiogenesis, invasion, and metastasis [19, 20]. The effects of caveolin-1 overexpression in various types of invasive tumor cells have also been investigated. Ho et al. [21] showed that caveolin-1 upregulation in lung adenocarcinoma cells mediated filopodia formation, which might enhance invasiveness. Recently, Yang et al. [22] reported that caveolin-1 was involved in the malignant progression of human prostate carcinoma via an interaction with c-Myc. In human scirrhous breast cancers, caveolin-1 mutation may activate cell invasiveness [23]. Additionally, caveolin-1 levels are elevated in metastatic or multidrug-resistant human cancer cell lines [24, 25]. Caveolin-1 expression is hypothesized to increase as cancer advances to promote tumor progression [26]. Moreover, the pathophysiological process underling the role of caveolin-1 has been already highlighted by a clinical point of view in studies indicating that indirect markers of portal hypertension are in turn associated with increased HCC occurrence [27] and recurrence [28]. In particular, as it has been reported, caveolin-1 level may be a good predictor for the efficacy of capecitabine/gemcitabine therapies and there is evidence of efficacy of metronomic capecitabine in HCC [29]. However, the mechanisms underlying caveolin-1-mediated promotion of tumor invasiveness and angiogenesis remain unclear.

Lentiviral vectors, which are known to effectively establish long-term transgene expression [30], were used to construct caveolin-1 siRNA-expressing cells. Using this approach, we confirmed that caveolin-1 mRNA and protein levels were significantly knocked down in SMMC7721 cells that had been infected with caveolin-1-siRNA-LV. In caveolin-1 siRNA-expressing cells, VEGF secretion was significantly reduced. Caveolin-1 RNAi also caused an inhibition of SMMC7721 cell proliferation and cell cycle progression that was accompanied by increased apoptosis. These results are consistent with other studies that demonstrated that secreted caveolin-1 promotes prostate cancer cell proliferation [31] and negatively regulates TRAIL-induced apoptosis in human hepatocarcinoma cells (HepG2) [32]. Invasion assay data revealed that caveolin-1 knockdown inhibited SMMC7721 invasiveness *in vitro*. Interestingly, supplementation with recombinant human VEGF restored SMMC7721 cell invasiveness. These results agree with a previous report [33] that demonstrated that caveolin-1 downregulation reduced lymphatic metastasis in mouse hepatocarcinoma cells. Thus, our results imply that caveolin-1 is involved in SMMC7721 cell invasiveness by regulating VEGF-induced angiogenesis.

To confirm the hypothesis, we performed *in vitro* three-dimensional Matrigel growth assays and *in vivo* tumorigenicity and angiogenesis induction assays. The Matrigel system is an established, reliable model used to assess angiogenesis *in vitro* [34, 35]. Short-term culture (≤24 h) on Matrigel does not involve cell proliferation or migration [36, 37]. Therefore, we employed the GFR Matrigel assay system to investigate the potential role of SMMC7721 caveolin-1 in endothelial cell differentiation. Our results clearly demonstrated that caveolin-1 RNAi-induced VEGF downregulation in SMMC7721 cells significantly reduced endothelial capillary tubule formation; furthermore, endothelial capillary formation was rescued following supplementation with recombinant human VEGF. These observations suggest that downregulation of caveolin-1 could prevent VEGF-induced endothelial capillary tubule formation. Consistent with these *in vitro* results, we showed that caveolin-1 siRNA markedly reduced SMMC7721 tumorigenicity and angiogenesis induction *in vivo*. Recently, clinical studies asserted a positive correlation between caveolin-1 expression, MVD, and poor prognosis in HCC [38–41] and prostate [42] cancer patients. These observations are consistent with the findings that caveolin-1 promotes SMMC7721 cell invasiveness through the regulation of VEGF-induced angiogenesis *in vitro* and *in vivo*.

## 5. Conclusions

Taken together, our data demonstrate that caveolin-1 knockdown inhibits SMMC7721 cell growth and proliferation, promotes apoptosis, and reduces VEGF secretion. Furthermore, downregulation of caveolin-1 inhibits SMMC7721 cell invasiveness by preventing VEGF-induced angiogenesis *in vitro* and *in vivo*. Our results are consistent with the hypothesis that caveolin-1 expression plays a critical role in HCC progression and angiogenesis.

## Data Availability

The data used to support the findings of this study are available from the corresponding author upon request.
